# Predictors of Intimate Partner Violence Among Reproductive Age Group Women in Mozambique

**DOI:** 10.1002/puh2.70350

**Published:** 2026-08-14

**Authors:** Russell Kabir, Haniya Zehra Syed, Ashek Elahi Noor, Angi Mohamed, Ali Davod Parsa

**Affiliations:** ^1^ School of Allied Health and Social Care Anglia Ruskin University Chelmsford UK; ^2^ School of Medicine Anglia Ruskin University Chelmsford UK; ^3^ Department of Computing, Healthcare Innovation and Technology Atlantic Technological University Galway Ireland; ^4^ Association of Public Health Research—Epidemiological Laboratory (Epi‐Lab) Khartoum Sudan

**Keywords:** intimate partner violence, Mozambique, predictors, reproductive age

## Abstract

**Background:**

Intimate partner violence (IPV) is a major public health and human rights issue globally, with a particularly high burden in sub‐Saharan Africa. Despite this, evidence on the predictors of IPV in Mozambique remains limited. This study aimed to identify predictors of IPV among women of reproductive age in Mozambique using nationally representative data.

**Methods:**

This study utilised secondary data from the 2022 to 2023 Mozambique Demographic and Health Survey (DHS). A total of 4454 women aged 15–49 years who responded to the domestic violence module were included. IPV was defined as experiencing at least one act of physical, sexual or emotional violence by a current or former intimate partner in the 12 months preceding the survey. Logistic regression, accounting for sampling weights, clustering and stratification, was used to identify independent predictors of IPV. Adjusted odds ratios (AORs) with 95% confidence intervals (CIs) were reported.

**Results:**

The prevalence of IPV among reproductive‐age women in Mozambique was 24.8% (1103/4454). In the adjusted model, higher education was associated with significantly reduced odds of IPV (AOR = 0.420, 95% CI: 0.243–0.725). Employment was associated with increased odds of IPV (AOR = 1.192, 95% CI: 1.022–1.390). Partner alcohol use was the strongest predictor (AOR = 2.078, 95% CI: 1.781–2.425), followed by a history of father‐to‐mother violence (AOR = 2.022, 95% CI: 1.681–2.432). Women in the richest wealth quintile had significantly lower odds of IPV (AOR = 0.631, 95% CI: 0.462–0.862).

**Conclusion:**

IPV remains highly prevalent among women in Mozambique and is strongly influenced by relational and intergenerational factors. Partner alcohol consumption and exposure to parental violence significantly increase the risk, whereas higher education offers protection. These findings highlight the need for integrated interventions addressing alcohol use, intergenerational violence and gender norms, alongside efforts to promote women's education and empowerment.

## Graphical abstract

Intimate partner violence affects 24.8% of women in Mozambique. Key risk factors include partner alcohol use, intergenerational violence and women's employment, whereas higher education, wealth and older age are protective. Findings highlight the need for integrated interventions targeting alcohol use, family violence and gender norms.

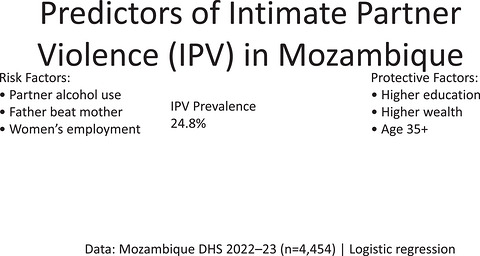

## 1 Introduction

Intimate partner violence (IPV), defined as any behaviour within an intimate relationship that causes physical, sexual or psychological harm, constitutes one of the most pervasive violations of women's human rights globally [1]. Nearly one in three women worldwide (30%) have experienced physical or sexual IPV or non‐partner sexual violence in their lifetime, with some sub‐Saharan African countries reporting rates as high as 40%–60% [2]. The consequences of IPV extend past immediate physical injury to include chronic mental health disorders, reproductive harm, increased HIV vulnerability, and high social and economic costs for families and communities [1, 3]. IPV is therefore not only a health burden but also a structural barrier to gender equality and sustainable development, directly undermining the United Nations Sustainable Development Goals, particularly Goals 5 on gender equality and 3 on good health and well‐being [4].

In sub‐Saharan Africa, the burden of IPV is disproportionately high and deeply entrenched. The prevalence of physical, emotional and sexual IPV in the region is approximately 30.58%, 30.22% and 12.6%, respectively. At least one form of IPV affects 42.62% of women in the region, representing the highest regional prevalence compared to other WHO regions [5]. Structural causes such as patriarchal gender norms, economic dependency, limited legal enforcement and culturally sanctioned attitudes toward wife‐beating sustain high violence rates [6]. Partner‐related factors lower educational attainment, alcohol consumption and controlling behaviour—increase the likelihood of IPV in sub‐Saharan African countries [6, 7]. Women in rural areas, those with lower educational attainment, and those in poorer wealth quintiles face increased risk. However, evidence increasingly shows that IPV transcends socioeconomic boundaries. Comprehensive and multi‐sectoral responses are required [8, 9, 10, 11].

Mozambique offers a particularly appropriate context for IPV research. The country is among the world's least developed, with high poverty rates, substantial gender inequality, widespread cohabitation and a post‐conflict legacy of social disruption. However, the epidemiology of IPV in Mozambique has received limited scholarly attention compared to neighbouring East African countries [12, 13, 14]. In Mozambique, IPV reflects deep‐rooted gender inequality and is often culturally accepted rather than challenged. For example, 45% of all injuries experienced by women in Maputo were related to domestic violence, with 78% perpetrated by a male partner [12]. Evidence consistently documents a high and persistent burden of IPV in Mozambique. Among women presenting to forensic services in Maputo, 70.2% had experienced one or more types of IPV in the preceding 12 months. Partner controlling behaviours and childhood abuse were identified as key explanatory factors [12]. Nationally, the 2011 Mozambique Demographic and Health Survey (DHS) found that 39.6% of married or in‐union women aged 15–49 had experienced some form of IPV in the past 12 months. Partner alcohol use, controlling behaviour and intergenerational exposure to parental violence were recurring correlates [15].

Although women's economic empowerment should be protective, its relationship with IPV is complex. Employment status was linked to a higher chance of experiencing lifetime physical IPV. Employed women had higher odds of IPV compared to those not employed. This paradox, seen across several sub‐Saharan contexts, may reflect how economic independence provokes controlling responses from male partners [16, 17]. Reproductive‐age women (15–49 years) are the main focus of IPV surveillance and intervention globally. They face greater exposure through union formation, pregnancy and childbearing [18, 19, 20]. In Tanzania, research using DHS data showed that IPV significantly reduces antenatal care use among ever‐married women [21]. Educational attainment emerged as a key moderating factor [21]. Similar dynamics were seen in India. Place of residence, educational status, employment status, age, partner's educational level and partner's alcohol consumption all significantly influenced women's decision‐making power and vulnerability to IPV [21, 22]. These findings are directly relevant to low‐income African contexts with similar sociodemographic profiles. Multi‐country analyses add support: Among four East African countries, including Mozambique, the pooled prevalence of IPV among reproductive‐age women was 40.18%. Mozambique showed the lowest estimate at 34.03%. This suggests relative, rather than absolute, protective factors warranting closer examination [23].

Despite more evidence from multi‐country DHS analyses and a few country‐specific studies, detailed epidemiological data on the determinants of IPV among reproductive‐age women in Mozambique remain underutilised, especially from earlier DHS waves. It is essential to identify which sociodemographic, attitudinal and relational factors independently predict IPV after thorough statistical adjustment. This targeting is key for public health interventions and national policy. This study uses data from the 2022–2023 Mozambique DHS to identify independent predictors of IPV among reproductive‐age women.

## 2 Methods

### 2.1 Data Source

This study is a cross‐sectional secondary data analysis utilising the most recent Mozambique DHS 2022–2023, conducted by the Instituto Nacional de Estatística (INE) in collaboration with ICF International under the DHS Programme. The DHS is a nationally representative household survey that collects comprehensive data on population health, nutrition, fertility and gender‐based violence indicators. The dataset was accessed with authorisation from the DHS Programme following their standard data request and approval procedure.

### 2.2 Sampling Process

The 2022–2023 Mozambique DHS employed a two‐stage stratified cluster sampling design. In the first stage, the DHS Programme selected 619 enumeration areas (EAs) from the 2017 Population and Housing Census using probability proportional to size sampling, with 232 from urban and 387 from rural areas. Eight districts in Cabo Delgado province were excluded due to security concerns. In the second stage, 26 households were systematically selected from each EA, yielding 16,045 sampled households. All women aged 15–49 who were either usual residents or visitors present on the night before the survey were eligible for interview. Data collection was conducted by trained DHS field teams following standardised DHS protocols. For the present secondary analysis, we used the domestic violence module sub‐sample, comprising 4454 women who completed the IPV questions.

### 2.3 Questionnaire

Five main questionnaires were used in the DHS 2022–2023: household, women aged 15–49, men aged 15–54, biomarker and water quality testing. These were based on the DHS Programme Model Questionnaires. For this study, variables from the Women's Questionnaire were used.

### 2.4 Key Variables and Data Analysis

IPV was the dependent variable, defined as a composite dichotomous variable (Yes/No). A respondent was classified as having experienced IPV if she reported at least one act of physical, sexual or emotional violence by a current or former husband/partner in the 12 months preceding the survey. Physical violence was assessed using DHS questions on whether the partner had ever pushed, shaken, slapped, punched, kicked, dragged, strangled, burned or threatened with a weapon (D105A–F, D105J). Sexual violence was assessed by questions on forced sexual intercourse and other forced sexual acts (D105H, D105I and D105K). Emotional violence was assessed by questions on humiliation, threats of harm and insults (D103A–C). Responses coded as 1 (often) or 2 (sometimes) indicated occurrence in the past 12 months. The DHS response categories for each item were 0 = not in the past 12 months, 1 = often, 2 = sometimes, 8 = don't know and 9 = not applicable. Responses coded as 1 or 2 indicated that the act had occurred in the past 12 months.

Specifically, responses of 1 (often) or 2 (sometimes) were treated as indicative of IPV occurrence (‘Yes’) for each item. Responses coded as 0 (not in past 12 months), 8 (don't know) or 9 (not applicable) were treated as the absence of that act. A respondent was classified as ‘No’ (not experiencing IPV) if she reported no acts coded as 1 or 2 across all physical, sexual and emotional violence items within the 12‐month reference period. The composite IPV variable was derived by combining all physical, sexual and emotional violence items: a woman was assigned IPV = ‘Yes’ if at least one item across any of the three domains was coded as 1 or 2 and IPV = ‘No’ otherwise.

The key independent variables of the study were

1. Place of residence, with two categories: urban and rural.
2. Highest educational level, with four categories: no education, primary, secondary and higher education.
3. Wealth index, measured in five categories: poorest, poorer, middle, richer and richest.
4. Age group, divided into three categories: 15–24, 25–34 and 35 years and above.
5. Employment status of the participants was categorised as ‘Yes’ (employed at the time of interview) and ‘No’ (not employed at the time of interview).
6. Pregnancy status, with two categories: (a) no or unsure and (b) yes.
7. Husband or family member pressured the respondent to become pregnant, with two categories: yes and no.
8. Marital status—six categories: never in union, married, living with partner, widowed, divorced and separated.
9. Partner's education level—five categories: no education, primary, secondary, higher and don't know.
10. Ability to refuse sex (*n* = 3320)—three categories: No, Yes and don't know.
11. Partner drinks alcohol—two categories: No, Yes.
12. Father ever beat mother—three categories: No, Yes and don't know.

The prevalence of IPV and background characteristics were described using frequencies and percentages. The chi‐square test was used to assess bivariate associations between each independent variable and IPV, with *p* < 0.05 considered statistically significant. Variables were selected for the multivariable model using a purposive approach guided by the ecological model framework and by statistical significance (*p* < 0.25) in bivariate analysis. Multicollinearity among independent variables was assessed using variance inflation factors (VIFs); all key predictor variables had VIFs below 2. Multivariable logistic regression was performed to identify independent predictors of IPV. Adjusted odds ratios (AORs) with 95% confidence intervals (CIs) were reported. Model fit was assessed using the Hosmer–Lemeshow goodness‐of‐fit test. Interaction terms (education × employment; partner alcohol use × residence) were tested. Statistical significance was established at *p* < 0.05 [24]. All analyses were performed using IBM SPSS Version 31.

## 3 Results

The overall prevalence of IPV among reproductive‐age women in Mozambique was 24.8% (1103/4454), indicating that approximately one in four women experienced at least one form of physical, sexual or emotional violence by an intimate partner in the 12 months preceding the survey. Table 1 presents the bivariate associations. The highest prevalence was observed among women aged 25–34 years (27.1%), those with primary education (26.7%), women in divorced unions (36.2%), women whose partners consumed alcohol (34.7%), and women who reported that their fathers had beaten their mothers (37.4%). Chi‐square tests indicated that age (*p* = 0.002), education (*p* = 0.001), religion (*p* < 0.001), marital status (*p* < 0.001), partner's education (*p* = 0.025), employment (*p* = 0.006), partner alcohol use (*p* < 0.001) and father's violence (*p* < 0.001) were significantly associated with IPV, whereas residence, wealth index, pregnancy status and ability to refuse sex were not.

**TABLE 1:** Frequency distribution and bivariate analysis of factors associated with intimate partner violence (IPV) among women of reproductive age in Mozambique (*n* = 4454).

Variable/Category	Total (*n*)	%	IPV = Yes	Prev. (%)	*χ* ^2^	*p* value
Age group					12.93	0.002
15–24	1513	34.0	387	25.6		
25–34	1488	33.4	403	27.1		
35+	1453	32.6	313	21.5		
Type of residence					0.81	0.369
Urban	1749	39.3	420	24.0		
Rural	2705	60.7	683	25.2		
Education level—respondent					15.9	0.001
No education	1189	26.7	278	23.4		
Primary	1929	43.3	515	26.7		
Secondary	1185	26.6	290	24.5		
Higher	151	3.4	20	13.2		
Religion					28.0	<0.001
Catholic	1105	24.8	242	21.9		
Islamic	860	19.3	187	21.7		
Zion	673	15.1	179	26.6		
Evangelical/Pentecostal	1376	30.9	367	26.7		
Anglican	90	2.0	16	17.8		
No religion	331	7.4	109	32.9		
Other	19	0.4	3	15.8		
Wealth index					7.12	0.130
Poorest	733	16.5	201	27.4		
Poorer	720	16.2	165	22.9		
Middle	889	20.0	229	25.8		
Richer	994	22.3	253	25.5		
Richest	1118	25.1	255	22.8		
Currently pregnant					0.4	0.529
No/Unsure	4053	91.0	998	24.6		
Yes	401	9.0	105	26.2		
Marital status					32.17	<0.001
Never in union	462	10.4	83	18.0		
Married	1239	27.8	321	25.9		
Living with partner	2081	46.7	541	26.0		
Widowed	162	3.6	23	14.2		
Divorced	116	2.6	42	36.2		
Separated	394	8.8	93	23.6		
Partner's education level					11.12	0.025
No education	696	21.0	168	24.1		
Primary	1306	39.3	353	27.0		
Secondary	856	25.8	234	27.3		
Higher	133	4.0	20	15.0		
Don't know	329	9.9	87	26.4		
Respondent currently working					7.68	0.006
No	2771	62.2	647	23.3		
Yes	1683	37.8	456	27.1		
Ability to refuse sex					4.42	0.110
No	1343	40.5	323	24.1		
Yes	1775	53.5	486	27.4		
Don't know	202	6.1	53	26.2		
Partner drinks alcohol					115.9	<0.001
No	2978	66.9	591	19.8		
Yes	1476	33.1	512	34.7		
Father ever beat mother					107.39	<0.001
No	3206	72.0	662	20.6		
Yes	685	15.4	256	37.4		
Don't know	563	12.6	185	32.9		

*Note:* IPV = past 12‐month experience.

Table 2 presents the unadjusted and adjusted logistic regression analyses. After adjustment, several factors remained independently associated with IPV. Higher education was protective (AOR = 0.420, 95% CI: 0.243–0.725, *p* = 0.002). Women aged 35+ had lower odds compared to 15–24‐year‐olds (AOR = 0.705, 95% CI: 0.583–0.853, *p* < 0.001). Employment was positively associated (AOR = 1.192, 95% CI: 1.022–1.390, *p* = 0.025). Women in the richest quintile had lower odds (AOR = 0.631, 95% CI: 0.462–0.862, *p* = 0.004). Having no religion was associated with higher odds (AOR = 1.496, 95% CI: 1.131–1.979, *p* = 0.005). The two strongest predictors were partner alcohol use (AOR = 2.078, 95% CI: 1.781–2.425, *p* < 0.001) and father beating mother (AOR = 2.022, 95% CI: 1.681–2.432, *p* < 0.001). The Hosmer–Lemeshow test confirmed adequate model fit (*χ* ^2^ = 7.234, df = 8, *p* = 0.512). Neither the education × employment (*χ* ^2^ = 1.870, *p* = 0.600) nor the alcohol × residence interaction (*χ* ^2^ = 3.465, *p* = 0.063) was significant.

**TABLE 2:** Unadjusted and adjusted logistic regression of factors associated with intimate partner violence (IPV) among women of reproductive age in Mozambique (*n* = 4454).

Variables	UOR	Lower	Upper	*p*	AOR	Lower	Upper	*p*
Age group								
15–24 (Ref.)								
25–34	1.202	1.043	1.386	0.011	0.986	0.831	1.17	0.873
35+	0.768	0.662	0.892	<0.001	0.705	0.583	0.853	<0.001
Residence								
Urban (Ref.)								
Rural	1.069	0.929	1.229	0.351	0.975	0.8	1.188	0.799
Education								
No education (Ref.)								
Primary	1.2	1.047	1.376	0.009	1.068	0.889	1.283	0.484
Secondary	0.979	0.839	1.142	0.786	0.926	0.721	1.189	0.547
Higher	0.454	0.282	0.73	0.001	0.42	0.243	0.725	0.002
Religion								
Catholic (Ref.)								
Islamic	0.812	0.68	0.971	0.023	1.114	0.889	1.395	0.349
Zion	1.121	0.93	1.35	0.231	1.067	0.842	1.351	0.592
Evangelical/Pentecostal	1.157	1.001	1.339	0.049	1.164	0.953	1.421	0.137
Anglican	0.652	0.378	1.124	0.124	0.722	0.405	1.286	0.268
No religion	1.546	1.216	1.965	<0.001	1.496	1.131	1.979	0.005
Other	0.568	0.165	1.955	0.370	0.696	0.197	2.46	0.574
Wealth index								
Poorest (Ref.)								
Poorer	0.886	0.734	1.071	0.211	0.774	0.606	0.989	0.040
Middle	1.069	0.903	1.265	0.441	0.822	0.651	1.038	0.100
Richer	1.049	0.891	1.233	0.567	0.768	0.594	0.994	0.045
Richest	0.867	0.739	1.017	0.080	0.631	0.462	0.862	0.004
Pregnant								
No/Unsure (Ref.)								
Yes	1.087	0.86	1.373	0.486	0.98	0.767	1.254	0.875
Employment								
No (Ref.)								
Yes	1.22	1.062	1.402	0.005	1.192	1.022	1.39	0.025
Partner alcohol								
No (Ref.)								
Yes	2.145	1.865	2.468	<0.001	2.078	1.781	2.425	<0.001
Father beat mother								
No (Ref.)								
Yes	2.059	1.732	2.447	<0.001	2.022	1.681	2.432	<0.001
Don't know	1.585	1.31	1.918	<0.001	1.736	1.419	2.123	<0.001

*Note:* Hosmer–Lemeshow *χ* ^2^ = 7.234, *p* = 0.512.

Abbreviation: AOR, adjusted odds ratio.

## 4 Discussion

This study examined the predictors of IPV among reproductive‐age women in Mozambique using nationally representative DHS data. The findings show that IPV is still a major public health and gender‐equity concern, with clear evidence that both individual and relational factors shape women's risk. In particular, higher educational attainment was associated with significantly reduced odds of IPV compared with no education, whereas primary and secondary education were not significantly associated with IPV. These findings suggest that the protective effect of education may become evident only at higher levels of educational attainment. Women's employment, partner alcohol use and exposure to intergenerational violence were associated with higher odds of IPV. The protective effect of higher education is consistent with evidence showing that women's schooling can improve autonomy, awareness of rights and capacity to negotiate within intimate relationships. Education may also expand access to information, social networks and resources that support help‐seeking and reduce tolerance of abuse. However, education alone is unlikely to eliminate IPV in settings where unequal gender norms remain deeply embedded. In contexts such as Mozambique, the benefits of education may be limited unless accompanied by broader structural and normative change [25, 26, 27].

The finding that employed women had higher odds of IPV is important and consistent with research from sub‐Saharan Africa showing that women's economic participation does not always translate into safety within the home [6, 7, 16]. Rather than serving as a straightforward protective factor, employment may sometimes provoke partner backlash when women's earning power questions traditional male authority [10, 28, 29]. This interpretation is especially plausible in settings where household decision‐making remains strongly gendered and where male partners may respond to women's economic independence with coercive control or violence. The result, therefore, suggests that economic empowerment initiatives should be paired with interventions that engage men and address harmful norms around masculinity, control and household power [16, 30]. Partner alcohol use emerged as one of the strongest predictors of IPV in this study. This finding is consistent with extensive research linking alcohol consumption to increased aggression, reduced judgement and conflict escalation in intimate relationships [22, 31, 32]. Alcohol use may also reveal broader patterns of risk‐taking, social stress or unstable household environments. From a policy perspective, this stresses the need for IPV prevention strategies that incorporate alcohol reduction and treatment services, especially in communities where harmful drinking is common and enforcement of protective laws is weak. Addressing alcohol use may be an especially feasible entry point for reducing IPV because it targets a modifiable behavioural risk factor [25, 27].

The association between exposure to father‐to‐mother violence and women's later experience of IPV strongly supports the intergenerational transmission of violence. Women who grow up in households where violence is normalised may be more likely to accept abuse as part of intimate relationships or may face elevated vulnerability due to damaged family functioning, trauma and learned patterns of conflict resolution. This finding is especially troubling because it suggests that IPV is not only a current health and social problem but also one that can be reproduced from generation to generation. Prevention efforts should therefore begin early in life and include family‐, school‐ and community‐based interventions that promote nonviolent conflict resolution and gender‐equitable norms [12, 33]. The lack of independent associations between IPV and wealth, residence or pregnancy status suggests that the drivers of IPV in Mozambique are not limited to socioeconomic disadvantage alone. Instead, the results point more strongly to relational power imbalances and behavioural risk factors. This is consistent with ecosystem models of violence, which emphasise that IPV is determined by the interaction of individual, relationship, community and societal determinants [12, 13, 14]. The findings also indicate that IPV cuts across social groups and cannot be assumed to occur only among poorer or rural women. As a result, prevention programmes should be designed to reach women across socioeconomic strata while also accounting for settings and groups facing additional vulnerabilities [16, 25]. The bivariate association between women's ability to refuse sex and IPV likely reflects complicated dynamics of autonomy, coercion and relationship control. Women who report greater sexual agency may be better able to recognise abuse, but refusal may likewise provoke conflict in relationships characterised by male dominance. The fact that this variable did not remain significant in the adjusted model suggests that its relationship with IPV may be mediated by other factors, particularly partner behaviour and household violence history. This draws attention to the need for future studies to examine the pathways through which sexual negotiation, control and coercion interact in intimate partnerships [27, 33].

Taken together, these findings show several effects on policy and practice. First, IPV prevention in Mozambique should prioritise partner alcohol use as a modifiable risk factor. Second, interventions must target intergenerational violence by supporting children and families exposed to domestic abuse. Third, women's empowerment initiatives should be integrated with gender‐transformative programming that confronts norms supporting male dominance and violence. Finally, health services, including reproductive and maternal health programmes, should routinely identify women at risk and provide safe referral pathways to psychosocial, legal and protection services [25, 26, 30, 34].

### 4.1 Limitations

This study has several limitations. First, the cross‐sectional design precludes causal inference. Second, IPV variables were self‐reported and may be subject to recall and social desirability bias. Third, residual confounding may persist due to unmeasured variables, such as partner employment, relationship duration and the number of children. Fourth, the ability to refuse sex variable was available only for currently married/cohabiting women (*n* = 3320), reflecting a DHS skip pattern. Fifth, the dataset extract did not contain DHS sampling weight variables (V005), so the current analysis is unweighted. Finally, the exclusion of eight districts in Cabo Delgado province may affect the representativeness of the sample. Despite these limitations, the nationally representative data and rigorous analytical approach strengthen the reliability of findings.

## 5 Conclusion

In conclusion, IPV among reproductive‐age women in Mozambique is strongly associated with women's employment, partner alcohol use and exposure to violence in the parental home, whereas higher education demonstrates a protective effect. These findings underscore the requirement for comprehensive IPV prevention strategies addressing women's circumstances, male partner behaviour, family violence histories and the gender norms that support abuse.

## Author Contributions

**Haniya Zehra Syed**: writing – original draft, writing – review and editing, data curation, resources, conceptualisation. **Angi Mohamed**: writing – review and editing, writing – original draft, software, resources, data curation. **Russell Kabir**: conceptualisation, investigation, writing – original draft, methodology, writing – review and editing, software, formal analysis, data curation, supervision. **Ali Davod Parsa**: conceptualisation, writing – original draft, writing – review and editing, methodology, formal analysis, data curation, supervision. **Ashek Elahi Noor**: methodology, writing – original draft, writing – review and editing, formal analysis, data curation, resources.

## Funding

The authors have nothing to report.

## Ethics Statement

This study was based on secondary analysis of anonymised data from the 2022 to 2023 Mozambique Demographic and Health Survey (DHS). The original survey received ethical approval from the ICF Institutional Review Board and the Comité Nacional de Bioética para a Saúde (CNBS) of Mozambique. Written informed consent was obtained from all participants before data collection. Permission to access and use the DHS dataset was obtained through the DHS Programme. As the present study analysed publicly available, de‐identified data, no additional ethical approval or informed consent was required.

## Conflicts of Interest

The authors declare no conflicts of interest.

## Data Availability

The data used in this study are publicly available from the Demographic and Health Surveys (DHS) Programme. The 2022–2023 Mozambique DHS dataset can be accessed upon registration and approval of a data request at https://dhsprogram.com.
